# Risikofaktoren für einen verlängerten Krankenhausaufenthalt nach elektiver minimalinvasiver Kolonchirurgie in Deutschland – Datenanalyse der StuDoQ-Register

**DOI:** 10.1007/s00104-026-02510-4

**Published:** 2026-05-26

**Authors:** M. Kaufmann, S. Hetjens, C. Klinger, C. Reißfelder, Julia Hardt

**Affiliations:** 1 Chirurgische Klinik, Universitätsmedizin Mannheim, Theodor-Kutzer-Ufer 1–3, 68167 Mannheim, Deutschland; 2 Medizinische Statistik, Biomathematik und Informationsverarbeitung, Universitätsmedizin Mannheim, Theodor-Kutzer-Ufer 1–3, 68167 Mannheim, Deutschland; 3 StuDoQ-Register, Deutsche Gesellschaft für Allgemein- und Viszeralchirurgie, Berlin, Deutschland

**Keywords:** Kolonkarzinom, Sigmadivertikulitis, Prädiktoren, Krankenhausverweildauer, Kolorektalchirurgie, Colon cancer, Sigmoid diverticulitis, Predictors, Length of hospital, Colorectal surgery

## Abstract

**Einleitung:**

Im Rahmen dieser Analyse wurde die Krankenhausverweildauer (VWD) der elektiven minimalinvasiven onkologischen Kolonresektionen und der Sigmaresektionen bei Sigmadivertikulitis zwischen 2013 und 2024 aus den entsprechenden StuDoQ-Registern untersucht. Das Ziel dieser Analyse war es, Risikofaktoren für eine verlängerte VWD zu identifizieren.

**Methodik:**

Es wurden retrospektiv die Daten aus den StuDoQ-Registern „Kolonkarzinom“ und „LapSigma“ untersucht. Notfalleingriffe, offene/endoskopische Eingriffe und konvertierte Operationen wurden ausgeschlossen. Die gemeinsamen Variablen der beiden Register waren das Alter, das Geschlecht, der Body-Mass-Index (BMI), die American Society of Anesthesiologists(ASA)-Klasse, die OP-Dauer sowie der Zugang bei OP-Beginn. Beim Kolonkarzinom wurden zusätzlich zum chirurgischen Eingriff die Lokalisation des Tumors, das pT-Stadium, das pN-Stadium und der M‑Status ausgewertet. Beim „LapSigma“-Register wurden die Indikation des Eingriffes und die Klassifikation untersucht. Es wurden eine uni- und multivariate Analyse durchgeführt.

**Ergebnisse:**

Im „Kolonkarzinom“-Register konnten zwischen 2013 und 2024 von 52.203 Patientinnen und Patienten 17.930 in die Analyse inkludiert werden. Die mediane VWD lag bei 8 Tagen (IQR 6–11). Es zeigte sich in der multivariaten Analyse, dass insbesondere die ASA-Klasse (Odds Ratio 1,44, CI95% [1,37–1,52], *p*<0,001) und der M‑Status (Odds Ratio 1,24, CI95% [1,24–1,34], *p*<0,001) Risikofaktoren für eine VWD über 8 Tagen waren. Zwischen 2013 und 2024 wurden im „LapSigma“-Register 33.669 Patientinnen und Patienten erfasst, von denen 26.324 Patientinnen und Patienten in die Analyse eingeschlossen werden konnten. Die mediane VWD lag bei 7 Tagen (IQR 6–8). In der multivariaten Analyse zeigte sich, dass vor allem die ASA-Klasse (Odds Ratio 1,35, CI95% [1,29–1,42], *p*<0,001) und die Indikation der Operation (Odds Ratio 1,62, CI95% [1,53–1,73], *p*<0,001) einen signifikanten Einfluss auf die VWD hatten.

**Diskussion:**

Im Rahmen der Analyse zeigte eine Vielzahl der untersuchten Variablen bei einer sehr hohen Fallzahl einen signifikanten Effekt auf. Bei näherer Betrachtung sind bei laparoskopischen Sigmaresektionen im Rahmen einer Sigmadivertikulitis eine höhere ASA-Klasse und Indikation der Operation (frühelektiv und elektiv bei Blutung) für eine verlängerte VWD ausschlaggebend. Bei den onkologischen Resektionen zeigten sich eine höhere ASA-Klasse und ein positiver M‑Status als signifikanter Risikofaktor für eine verlängerte VWD.

**Graphic abstract:**

**Figure:**
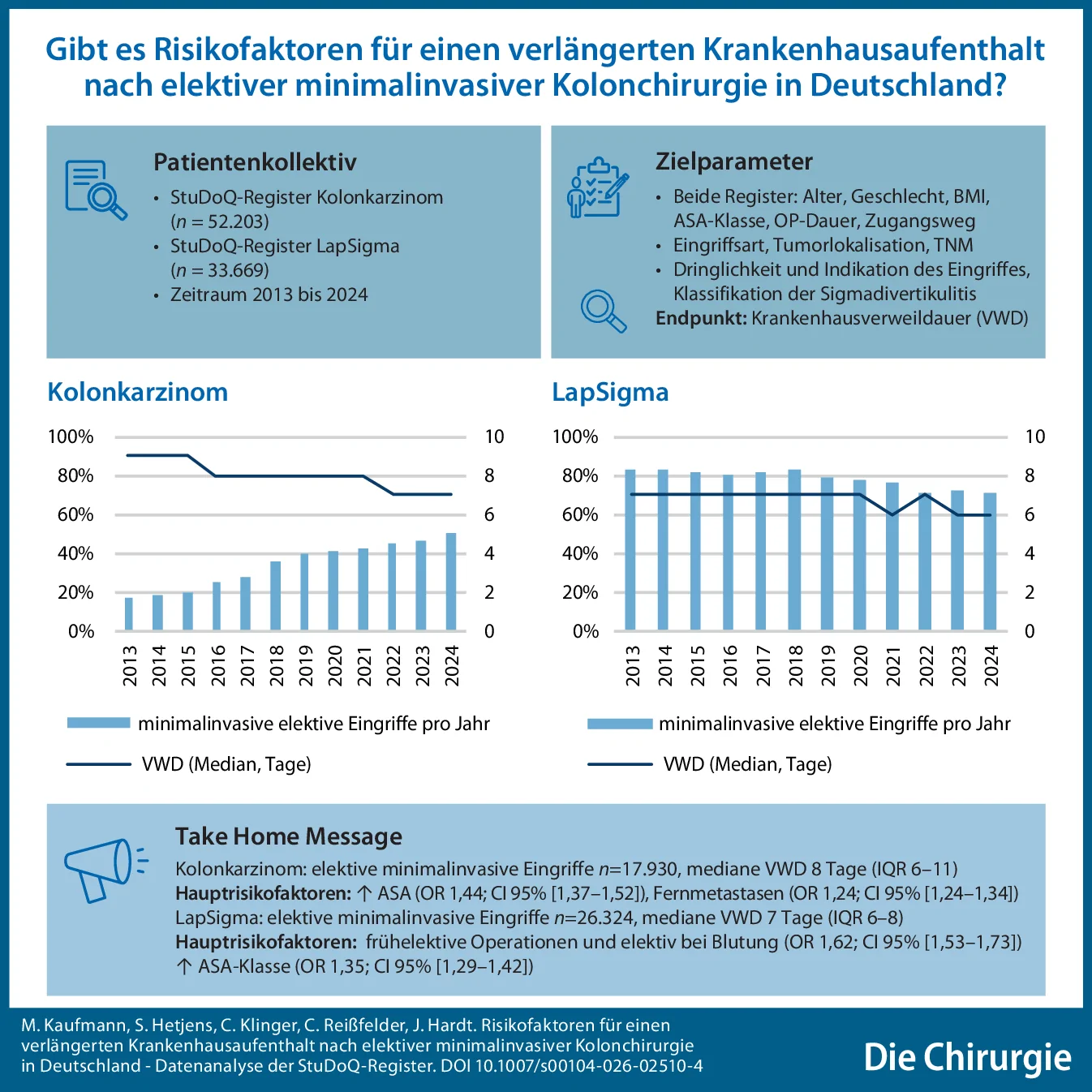

**Zusatzmaterial online:**

Die Online-Version dieses Beitrags (https://doi.org/10.1007/s00104-026-02510-4) enthält die Angaben aller Kliniken, die ihre Daten in die analysierten StuDoQ-Register eingeben.

## Abstract (en)

**Introduction:**

Within the framework of this analysis the duration of the hospital stay of elective minimally invasive oncological colon resections and sigma resections in cases of sigmoid diverticulitis between 2013 and 2024 from the corresponding StuDoQ registers were investigated. The aim of the analysis was to identify risk factors for an extended duration of the hospital stay.

**Method:**

The data from the StuDoQ registers “Colon cancer” and “LapSigma” were examined. Emergency interventions, open/endoscopic interventions and converted operations were excluded. The shared variables of both registers were age, sex, body mass index (BMI), the American Society of Anesthesiologists (ASA) class, the duration of surgery and the access at the start of surgery. For colon cancer the localization of the tumor, the pT stage, the pN stage and the M status were evaluated in addition to the surgical intervention. For the “LapSigma” register the indications for the intervention and the classification were investigated. Univariate and multivariate analyses were carried out.

**Results:**

In the “Colon cancer” register between 2013–2024 from 52,203 patients 17,930 could be included in the analysis. The median duration of hospital stay was 8 days (IQR 6–11). The multivariate analysis showed that the ASA class (odds ratio 1.44, 95% CI 1.37–1.52, *p*<0.001) and the M status (odds ratio 1.24, 95% CI 1.24–1.34, *p*<0.001) were risk factors for a hospital stay lasting longer than 8 days. In the “LapSigma” register between 2013 and 2024 there were a total of 33,669 patients of whom 26,324 patients could be included in the analysis. The median duration of hospital stay was 7 days (IQR 6–8). The multivariate analysis showed that the ASA class (odds ratio 1.35, 95% CI 1.29–1.42, *p*<0.001) and the indications for surgery (odds ratio 1.62, 95% CI 1.53–1.73,* p*<0.001) had a significant influence on the duration of hospital stay.

**Discussion:**

Within the framework of the analysis many of the variables investigated showed a significant effect with a very high number of cases. Under closer examination, particularly for laparoscopic sigma resections for a sigmoid diverticulitis, a higher ASA class and indications for surgery (early elective and elective with bleeding) are decisive for an extended duration of hospital stay. For oncological resections a higher ASA class and a positive M status in particular were shown to be significant risk factors for an extended hospital stay.

## Einleitung

Das kolorektale Karzinom ist eine der häufigsten Tumorentitäten weltweit und ist beispielswiese in den USA die zweithäufigste krebsbedingte Todesursache [1]. In Deutschland sind sowohl Inzidenz als auch die Mortalität seit mehreren Jahren rückläufig, jedoch ist das kolorektale Karzinom mit etwa 55.000 Neuerkrankungen und ca. 23.000 Todesfällen im Jahr 2022 noch immer eine der häufigsten Tumorentitäten in Deutschland [2]. Hiervon gehen ca. zwei Drittel der Neuerkrankungen auf das Kolonkarzinom zurück. Das kolorektale Karzinom ist mit einem mittleren Erkrankungsalter bei Frauen mit 75 Jahren und bei Männern mit 71 Jahren eine Erkrankung des höheren Lebensalters [2]. Auch bei der Sigmadivertikulitis handelt es sich um eine Erkrankung der älteren Bevölkerung, da die Prävalenz einer Divertikulose, welche zu einer akuten Sigmadivertikulitis führen kann, mit dem Alter ansteigt. Während die Prävalenz bei unter 50-Jährigen bei etwa 13 % liegt, so ist bei 50 % der 50- bis 70-Jährigen eine Divertikulose vorhanden [3]. Patienten, die bereits einmalig eine Divertikulitis erlebten, erleiden mit 20 %iger Wahrscheinlichkeit ein Rezidiv innerhalb eines Jahres [4].

Bei beiden Krankheitsbildern stellt die elektive Chirurgie die zentrale Therapie dar [2]. Gemäß der aktuellen S3-Leitlinie „Divertikelkrankheit/Divertikulitis“ kann eine elektive Operation bei Vorliegen einer chronisch rezidivierenden Divertikulitis CDD Typ 3b – je nach Beeinträchtigung der Lebensqualität – erfolgen. Bei einer chronisch rezidivierenden Divertikulitis Typ 3c besteht ebenfalls eine OP-Indikation [3].

Unabhängig von der Entität spielt eine optimale perioperative Therapie eine entscheidende Rolle für eine schnelle Erholung der Patientinnen und Patienten nach der Operation. Entscheidend hierfür ist ein erfolgreiches perioperatives Management gemäß den ERAS®-Guidelines und im deutschsprachigen Raum gemäß der POMGAT-Leitlinie [5, 6]. Wesentlich für eine rasche Erholung nach einer Kolonresektion ist dabei eine minimalinvasive Operation, die auch bei Vorliegen eines Karzinoms mit dem gleichen onkologischen Ergebnis durchgeführt werden kann [7]. Nach erfolgreicher Therapie sollten die Patienten rasch mobilisiert und ein früher Kostaufbau sollte angestrebt werden [5, 6]. Durch ein optimales perioperatives Management lässt sich die Krankenhausverweildauer um 30–50 % verkürzen [8, 9].

Ziel dieser Analyse der StuDoQ-Register ist es, Risikofaktoren für eine verlängerte VWD nach elektiver minimalinvasiver Kolonchirurgie zu detektieren.

## Methodik

Im Rahmen dieser retrospektiven Analyse wurde in Zusammenarbeit mit der DGAV und dem StuDoQ-Register ein Export der Daten aus den Registern „Kolonkarzinom“ und „LapSigma“ durchgeführt. Dabei wurden zunächst beide Register auf den kleinsten gemeinsamen Nenner hin überprüft, um eine vergleichbare Datengrundlage zu schaffen. Beim Abgleich der Codebooks wurden folgende Variablen identisch in den Registern erfasst: Geburtsdatum, Geschlecht, Größe (cm), Gewicht (kg), Operationsdatum, American Society of Anesthesiologists(ASA)-Klasse und die postoperative Krankenhausverweildauer (VWD). Gesondert wurde im „Kolonkarzinom“-Register das Operationsverfahren des definitiven chirurgischen Eingriffs erfasst, welches im „LapSigma“-Register definitionsgemäß eine Sigmaresektion darstellte. Der Zugang bei OP-Beginn umfasste beim „Kolonkarzinom“ zudem robotische Eingriffe und beim „LapSigma“-Register Single-Port-Operationen. Zur Klassifikation der Erkrankungen wurde beim „Kolonkarzinom“ die Lokalisation des Karzinoms und das pT, pN und der M‑Status bei Entlassung erfasst. Beim „LapSigma“-Register wurden die Patienten in der StuDoQ-Datenbank gemäß der alten Klassifikation nach Hansen und Stock eingeteilt.

### Ein- und Ausschlusskriterien

#### StuDoQ-Register „Kolonkarzinom“

In die Auswertung wurden nur Patientinnen und Patienten eingeschlossen, bei denen keine Konversion, sondern eine elektive minimalinvasive chirurgische Therapie durchgeführt wurde. Es wurden daher Fälle ausgeschlossen, die endoluminal (koloskopisch, offen transanal, TEM/TEO) oder offen operiert wurden. Fälle, bei denen keine klaren Angaben (k. A., sonstiger Zugang oder unbekannt) zum Zugangsweg gemacht wurde, wurden ebenfalls exkludiert. Bei der OP-Technik wurden nur onkologische Kolonresektionen eingeschlossen, sodass endoluminale Verfahren (einfache Polypektomie, EMR, ESD, Vollwandresektion) und palliative Operationen (Portanlage, Stomaanlage, Umgehungsanastomosen, andere palliative Verfahren) ausgeschlossen wurden. Ebenfalls wurden Fälle ausgeschlossen, bei denen die Operationstechnik nicht genauer klassifiziert wurde.

#### StuDoQ-Register „LapSigma“

Beim „LapSigma“-Register wurden die notfallmäßigen und dringlichen Eingriffe ausgeschlossen. Zudem wurde Fälle ohne klare Definition des Zugangsweges (andere, NOTES) ausgeschlossen.

### Statistische Auswertung

Die statistische Analyse wurde mit der Software SAS 9.4 (SAS Institute, Cary, NC, USA) durchgeführt. Alle normalverteilten Variablen wurden mit dem t‑Test für Mittelwerte verglichen. Der χ^2^-Test wurde verwendet, um die Häufigkeiten zwischen den Gruppen zu vergleichen. Für die Abschätzung von Zusammenhängen von Risikofaktoren und der VWD wurden eine univariate und multivariate logistische Regression durchgeführt. Die Ergebnisse werden als Odds Ratio (OR) mit 95 %-Konfidenzintervall dargestellt. Es wurde durchgehend ein zweiseitiges Signifikanzniveau von *p* = 0,05 verwendet.

## Ergebnisse

### StuDoQ-Register „Kolonkarzinom“

Zwischen 2013 und 2024 wurden 52.203 Patientinnen und Patienten in das Register aufgenommen. Hiervon wurden 49.607 Patientinnen und Patienten in kurativer Intention operiert. Aus dieser Kohorte wurden 17.930 Patientinnen und Patienten elektiv und minimalinvasiv operiert und in die Analyse inkludiert. Über die vergangene Dekade hinweg konnte dabei beobachtet werden, dass der Anteil minimalinvasiver elektiver Eingriffe von initial 17 % im Jahr 2013 auf 50 % im Jahr 2024 angestiegen ist (Tab. 1). Gleichzeitig sind sowohl die mediane als auch mittlere VWD kontinuierlich über die Jahre abgefallen. Zwischen 2013 mit 9 (7–11) Tagen und 2024 mit 7 (5–9) Tagen kam es zu einer signifikanten Reduktion der medianen VWD (*p* ≤ 0,001). Über den gesamten Zeitraum hinweg lag die mediane VWD bei 8 Tagen (6 bis 11 Tagen) und die mittlere VWD bei 9,85 Tagen (± 8,66 Tagen) (Tab. 1).

**Tab. 1:** Krankenhausverweildauer des Registers „Kolonkarzinom“ 2013 bis 2024

Jahr	Gesamtzahl der Patienten (*n*) pro Jahr	Minimalinvasive elektive Eingriffe (*n*) pro Jahr	Median (Q1–Q3) (Tage)	Mittelwert (±SD) (Tage)
2013	1985	337 (17 %)	9 (7–11)	11,27 (± 9,38)
2014	2785	490 (18 %)	9 (7–12)	10,95 (± 8,48)
2015	3337	674 (20 %)	9 (7–12)	10,96 (± 7,86)
2016	3759	946 (25 %)	8 (7–12)	10,52 (± 6,83)
2017	4344	1188 (27 %)	8 (7–11)	10,58 (± 8,24)
2018	4573	1606 (35 %)	8 (7–11)	10,28 (± 8,2)
2019	4984	1936 (39 %)	8 (6–11)	10,13 (± 8,27)
2020	4850	2011 (41 %)	8 (6–10)	9,58 (± 7,1)
2021	5097	2153 (42 %)	8 (6–11)	9,82 (± 7,92)
2022	4909	2210 (45 %)	7 (6–10)	9,38 (± 7,24)
2023	4823	2237 (46 %)	7 (6–10)	9,23 (± 7,62)
2024	4260	2142 (50 %)	7 (5–9)	9,15 (± 13,41)
2013–2024		17.930	8 (6–11)	9,85 (± 8,66)

Q1 = 1. Quartil, Q3 = 2. Quartil

*SD* Standardabweichung

### Binomiale Analyse

Zu weiteren Analysen wurden die vordefinierten Charakteristika anhand des Medians von 8 Tagen berechnet, sodass 10.843 (60 %) Patientinnen und Patienten mit einer VWD von ≤ 8 Tagen mit 7087 Patientinnen und Patienten mit einer VWD von > 8 Tagen verglichen wurden (Tab. 2).

**Tab. 2:** Binomiale Analyse der Krankenhausverweildauer des Registers „Kolonkarzinom“ 2013 bis 2024

	≤ 8 (*n* = 10.843, 60 %)	> 8 (*n* = 7087, 40 %)	*p*-Wert
*Alter (Jahre) *(Mittelwert, SD)	67,95 (± 12,31)	72,72 (± 11,65)	< 0,001*
*BMI (kg/m*^*2*^*) *(Mittelwert, SD)	26,65 (± 4,94)	26,99 (± 5,3)	< 0,001*
*OP-Dauer in min *(Mittelwert, SD)	175,22 (± 66,72)	184,72 (± 76,71)	< 0,001*
*Geschlecht*			
Männer (*n* = 9363)	5657 (60 %)	3706 (40 %)	0,874**
Frauen (*n* = 8567)	5186 (61 %)	3381 (39 %)	
*ASA-Klasse*			
ASA 1 (*n* = 1354)	1029 (76 %)	325 (24 %)	< 0,001**
ASA 2 (*n* = 8701)	5771 (66 %)	2930 (34 %)	
ASA 3 (*n* = 7514)	3914 (52 %)	3600 (48 %)	
ASA 4 (*n* = 230)	129 (36 %)	230 (64 %)	
ASA 5 (*n* = 1)	0 (0 %)	1 (100 %)	
ASA 6 (*n* = 1)	0 (0 %)	1 (100 %)	
*Zugang bei OP-Beginn*			
Laparoskopische Präparation (*n* = 13.556)	8202 (61 %)	5354 (39 %)	< 0,001**
Laparoskopische Präparation handassistiert (*n* = 2606)	1477 (57 %)	1129 (43 %)	
Robotik-assistiert (*n* = 1768)	1164 (66 %)	604 (34 %)	
*Operationsverfahren*			
Erweiterte Hemikolektomie links (*n* = 416)	205 (49 %)	211 (51 %)	< 0,001**
Erweiterte Hemikolektomie rechts (*n* = 1071)	558 (53 %)	512 (48 %)	
Hemikolektomie links (*n* = 2027)	1239 (61 %)	788 (39 %)	
Hemikolektomie rechts (*n* = 7221)	4388 (61 %)	2833 (39 %)	
Kolektomie (*n* = 167)	58 (35 %)	109 (65 %)	
Multiviszeralresektion (*n* = 39)	18 (46 %)	21 (54 %)	
Proktokolektomie (*n* = 129)	40 (31 %)	89 (69 %)	
Rektosigmoidresektion (*n* = 2204)	1375 (62 %)	829 (38 %)	
Sigmaresektion (*n* = 4265)	2735 (64 %)	1530 (36 %)	
Transversumresektion (*n* = 391)	226 (58 %)	165 (46 %)	
*Lokalisation des Karzinoms*			
C18,0: Zäkum (*n* = 2759)	1632 (59 %)	1127 (41 %)	< 0,001**
C18,1: Appendix vermiformis (*n* = 144)	106 (74 %)	38 (26 %)	
C18,2: Colon ascendens (*n* = 3946)	2401 (61 %)	1545 (39 %)	
C18,3: rechte Kolonflexur (*n* = 1062)	614 (58 %)	448 (42 %)	
C18,4: Colon transversum (*n* = 1009)	517 (51 %)	492 (49 %)	
C18,5: linke Kolonflexur (*n* = 494)	252 (51 %)	242 (49 %)	
C18,6: Colon descendens (*n* = 1027)	610 (59 %)	417 (41 %)	
C18,7: Colon sigmoideum (Sigma) (*n* = 6917)	4391 (63 %)	2526 (37 %)	
C18,8: Kolon, mehrere Teilbereiche überlappend (*n* = 65)	35 (54 %)	30 (46 %)	
C18,9: Kolon, nicht näher bezeichnet (*n* = 49)	31 (63 %)	18 (37 %)	
C19: rektosigmoidaler Übergang (*n* = 455)	253 (56 %)	202 (44 %)	
k. A. (*n* = 3)	1 (33 %)	2 (67 %)	
*pT*			
k. A. (*n* = 4)	1 (25 %)	3 (75 %)	< 0,001**
pT0 (*n* = 204)	125 (61 %)	79 (39 %)	
pT1 (*n* = 2798)	1846 (66 %)	952 (34 %)	
pT2 (*n* = 3658)	2285 (62 %)	1373 (38 %)	
pT3 (*n* = 9181)	5468 (60 %)	3713 (40 %)	
pT4a (*n* = 1622)	890 (55 %)	732 (45 %)	
pT4b (*n* = 392)	175 (45 %)	217 (55 %)	
pTX (*n* = 71)	53 (75 %)	18 (25 %)	
*pN*			
k. A. (*n* = 7)	3 (43 %)	4 (57 %)	0,001**
pN0 (*n* = 11.910)	7331 (62 %)	4579 (38 %)	
pN1a (*n* = 1901)	1152 (61 %)	749 (39 %)	
pN1b (*n* = 1707)	978 (57 %)	729 (43 %)	
pN1c (*n* = 258)	152 (59 %)	106 (41 %)	
pN2a (*n* = 1082)	628 (58 %)	454 (42 %)	
pN2b (*n* = 1019)	572 (56 %)	447 (44 %)	
pNX (*n* = 46)	27 (59 %)	19 (41 %)	
*M‑Status bei Entlassung*			
k. A. (*n* = 8)	3 (38 %)	5 (62 %)	< 0,001**
M0 (*n* = 16.272)	9976 (61 %)	6296 (39 %)	
M1a (*n* = 1087)	602 (55 %)	485 (45 %)	
M1b (*n* = 563)	262 (47 %)	301 (53 %)	

*SD* Standardabweichung

*t-Test, **χ^2^-Test

Es zeigte sich, dass eine kurze VWD signifikant mit einem jüngeren Alter von 68 Jahren (± 12,31) im Vergleich zu 73 Jahren (± 11,65) verbunden war (*p* < 0,001). Zudem war der BMI mit 26,65 (± 4,94) signifikant geringer in der Kohorte mit einer kürzeren VWD als in der Gruppe mit einer längeren VWD mit 26,99 (± 5,3) (*p* < 0,001). Bei Patienten mit einer kürzeren VWD war darüber hinaus die OP-Dauer mit 175 min (± 66,72 min) signifikant kürzer als bei Patienten mit einer längeren VWD mit 185 min (± 76,71) (*p* < 0,001) (Tab. 2).

Das Geschlecht hatte keinen Einfluss auf die Länge des Krankenhausaufenthaltes. Eine ASA-Klasse ≥ 4 war hingegen ein Risikofaktor für eine VWD über 8 Tagen (*p* < 0,001). Ein signifikanter Einfluss zeigte sich ebenfalls beim OP-Zugang, da die Patientinnen und Patienten mit einer „laparoskopischen Präparation handassistiert“ eher eine längere VWD aufwiesen (*p* < 0,001). Bei den operativen Eingriffen war die VWD am längsten, wenn bei den Eingriffen mehr als ein Kolonsegment entfernt wurde (Kolektomie, Proktokolektomie, Multiviszeralresektion) (*p* < 0,001). Zudem hatte die Lokalisation des Karzinoms einen signifikanten Einfluss auf die VWD (*p* < 0,001). Auch das T‑Stadium (ab T3), der Nodalstatus (ab pN1b) und eine vorliegende Metastasierung waren signifikante Risikofaktoren für eine verlängerte VWD (*p* < 0,001; 0,001; *p* < 0,001) (Tab. 2).

### Multivariate Analyse

In der multivariaten Analyse zeigten sich das Alter (OR 1,03, CI95 % 1,03–1,03, *p* < 0,001), der BMI (OR 1,01, CI95 % 1,01–1,02, *p* < 0,001), die OP-Dauer (OR 1,00, CI95 % 1,00–1,00, *p* < 0,001), die ASA-Klasse (OR 1,44, CI95 % 1,37–1,52, *p* < 0,001), der Zugang bei OP-Beginn (OR 1,06, CI95 % 1,04–1,08, *p* < 0,001), das Operationsverfahren (OR 1,01, CI95 % 1,01–1,02, *p* = 0,001), die Tumorlokalisation (OR 1,02, CI95 % 1,01–1,04, *p* = 0,001), das pT-Stadium (OR 1,07, CI95 % 1,03–1,11, *p* < 0,001), der Nodalstatus (OR 1,03, CI95 % 1,00–1,05, *p* = 0,028) und die Metastasierung (OR 1,24, CI95 % 1,14–1,34, *p* < 0,001) als signifikante Einflussfaktoren für eine VWD > 8 Tage. Das Geschlecht hatte keinen Einfluss (Tab. 3).

**Tab. 3:** Multivariate Analyse der Krankenhausverweildauer des Registers „Kolonkarzinom“ 2013 bis 2024

	Odds Ratio (CI95 %)	*p*-Wert
*Alter*	1,03 (1,03–1,03)	< 0,001
*BMI*	1,01 (1,01–1,02)	< 0,001
*OP-Dauer*	1,00 (1,00–1,00)	< 0,001
*Geschlecht*	1,03 (0,98–1,1)	0,357
*ASA*	1,44 (1,37–1,52)	< 0,001
*Zugang bei OP-Beginn*	1,06 (1,04–1,08)	< 0,001
*Operationsverfahren*	1,01 (1,01–1,02)	0,001
*Tumorlokalisation*	1,02 (1,01–1,04)	0,001
*pT*	1,07 (1,03–1,11)	< 0,001
*pN*	1,03 (1,00–1,05)	0,028
*M‑Status bei Entlassung*	1,24 (1,14–1,34)	< 0,001

### StuDoQ-Register „LapSigma“

Im StuDoQ-Register „LapSigma“ konnten zwischen 2013 und 2014 33.669 Patientinnen und Patienten erfasst werden. Aus diesem Kollektiv konnten nach Prüfung der Ein- und Ausschlusskriterien 26.324 Patientinnen und Patienten in diese Analyse eingeschlossen werden. Die mediane VWD ist in der letzten Dekade von 7 Tagen (6–9) im Jahr 2013 auf 6 Tage (5–8) im Jahr 2024 signifikant gefallen (*p* = 0,001). Die mediane VWD lag zwischen 2013 und 2024 bei 7 Tagen (6–8) und die mittlere VWD bei 8 Tagen (± 5,33). Über die Jahre hinweg ist der Prozentsatz von minimalinvasiven elektiven Eingriffen von 83 % im Jahr 2013 auf 71 % im Jahr 2013 gefallen (Tab. 4).

**Tab. 4:** Krankenhausverweildauer des Registers „LapSigma“ 2013 bis 2024

Jahr	Gesamtzahl der Patienten (*n*) pro Jahr	Minimalinvasive elektive Eingriffe (*n*) pro Jahr	Median (Q1–Q3) (Jahre)	Mittelwert (±SD) (Jahre)
2013	1999	1668 (83 %)	7 (6–9)	8,4 (± 4,58)
2014	2348	1938 (83 %)	7 (6–9)	8,72 (± 5,76)
2015	2447	2018 (82 %)	7 (6–9)	8,48 (± 5,1)
2016	2904	2332 (80 %)	7 (6–9)	8,48 (± 5,43)
2017	3109	2533 (81 %)	7 (6–9)	8,4 (± 5,27)
2018	3154	2604 (83 %)	7 (6–9)	8,38 (± 6,15)
2019	3237	2549 (79 %)	7 (6–8)	8,08 (± 5,37)
2020	2984	2283 (77 %)	7 (6–8)	7,98 (± 5,65)
2021	3693	2806 (76 %)	6 (5–8)	7,57 (± 5,17)
2022	2311	1650 (71 %)	7 (5–8)	7,58 (± 4,93)
2023	2823	2044 (72 %)	6 (5–8)	7,5 (± 4,93)
2024	2660	1885 (71 %)	6 (5–8)	7,27 (± 4,66)
2013–2024		26.324	7 (6–8)	8,08 (± 5,33)

Q1 = 1. Quartil, Q3 = 2. Quartil

*SD* Standardabweichung

### Binomiale Analyse

Zur weiteren Analyse wurde eine VWD ≤ 7 (*n* = 16.498, 63 %) mit einer VWD 7 (*n* = 9826, 37 %) verglichen. Es zeigte sich, dass in der Kohorte mit einer VWD ≤ 7 Tagen die Patientinnen und Patienten mit 59 Jahren (± 11,25) signifikant jünger waren als in der Kohorte mit einer VWD von > 7 Tagen mit 63 Jahren (± 12,35) (*p* < 0,001). Ebenfalls war ein niedrigerer BMI mit 27,43 (± 4,91) im Vergleich zu 27,62 (± 5,28) mit einer kürzeren VWD assoziiert (*p* = 0,003). Patientinnen und Patienten, die innerhalb einer Woche das Krankenhaus verließen, wurden mit 144 min (± 59,6) signifikant kürzer operiert als Patienten, die länger als eine Woche im Krankenhaus verblieben, mit 160 min (± 67,21) (*p* < 0,001) (Tab. 5).

**Tab. 5:** Binomiale Analyse der Krankenhausverweildauer des Registers „LapSigma“ 2013 bis 2024

	≤ 7 (*n* = 16.498, 63 %)	> 7 (*n* = 9826, 37 %)	*p*-Wert
*Alter (Jahre) *(Mittelwert, SD)	58,58 (± 11,25)	62,8 (± 12,35)	< 0,001*
*BMI (kg/m*^*2*^*) *(Mittelwert, SD)	27,43 (± 4,91)	27,62 (± 5,28)	0,003*
*OP-Dauer in min *(Mittelwert, SD)	143,6 (± 59,6)	160,08 (± 67,21)	< 0,001*
*Geschlecht*			
Männer (*n* = 11.186)	7246 (65 %)	3940 (35 %)	< 0,001**
Frauen (*n* = 15.138)	9252 (61 %)	5886 (39 %)	
*ASA-Klasse*			
ASA 1 (*n* = 3922)	2796 (71 %)	1126 (29 %)	< 0,001**
ASA 2 (*n* = 17.108)	11.167 (65 %)	5941 (35 %)	
ASA 3 (*n* = 5176)	2495 (48 %)	2681 (52 %)	
ASA 4 (*n* = 106)	31 (29 %)	75 (71 %)	
ASA 5 (*n* = 12)	9 (75 %)	3 (25 %)	
*Indikation der Operation*			
Elektiv bei Sigmadivertikelblutung (*n* = 136)	56 (41 %)	80 (59 %)	< 0,001**
Elektiv bei Z. n. Sigmadivertikulitis (> 14 Tage nach Symptombeginn bei akuter Divertikulitis) (*n* = 19.266)	12.737 (66 %)	6529 (34 %)	
Frühelektiv bei akuter Sigmadivertikulitis (innerhalb 14 Tage nach Symptombeginn) (*n* = 6922)	3705 (54 %)	3217 (46 %)	
*Zugang bei OP-Beginn*			
Laparoskopisch (ohne Bergeinzision) (*n* = 1501)	951 (63 %)	550 (37 %)	0,379**
Laparoskopisch assistiert (mit Bergeinzision > 2 cm) (*n* = 24.191)	15.166 (63 %)	9025 (47 %)	
Single Port (*n* = 632)	381 (60 %)	251 (40 %)	
*Klassifikation nach Hansen und Stock*			
Stadium I (akute unkomplizierte Divertikulitis) (*n* = 515)	323 (63 %)	192 (37 %)	< 0,001**
Stadium IIa (Peridivertikulitis, phlegmonöse Divertikulitis) (*n* = 1776)	1125 (63 %)	651 (37 %)	
Stadium IIb (abszedierende Divertikulitis, gedeckte Perforation, Fistel) (*n* = 10.642)	6332 (60 %)	4310 (40 %)	
Stadium IIc (freie Perforation) (*n* = 190)	112 (59 %)	78 (41 %)	
Stadium III (chronisch rezidivierende Divertikulitis) (*n* = 12.174)	7979 (66 %)	4195 (34 %)	
Patient wurde nicht klassifiziert (*n* = 1027)	627 (39 %)	400 (39 %)	

*SD* Standardabweichung

*t-Test, **χ^2^-Test

In der Gruppe mit einer Sigmadivertikulitis verweilten signifikant mehr Frauen (39 %) als Männer (35 %) länger als 7 Tage im Krankenhaus (*p* < 0,001). Die ASA-Klasse (≥ 4 ASA) hatte ebenfalls einen Einfluss auf die VWD (*p* < 0,001). Bei der Indikation der Operation zeigte sich, dass insbesondere die Patientinnen und Patienten, welche „elektiv bei Sigmadivertikelblutung“ operiert wurden, länger im Krankenhaus verweilten (*p* ≤ 0,001). Die Art des minimalinvasiven Operationszugangs hatte keinen Einfluss auf die VWD. Bei der Klassifikation nach Hansen und Stock lag die VWD insbesondere bei Stadium IIb und IIC über 7 Tage (*p* < 0,001) (Tab. 5).

### Multivariate Analyse

Unter Berücksichtigung aller Einflussfaktoren in der multivariaten Analyse zeigte sich, dass das Alter (OR 1,03, CI95 % 1,02–1,03, *p* ≤ 0,001), der BMI (OR 1,00,CI95 % 1,00–1,01, *p* = 0,031), die OP-Dauer (OR 1,00, CI95 % 1,00–1,00, *p* < 0,001), die ASA-Klasse (OR 1,35, CI95 % 1,29–1,42, *p* < 0,001) und die Indikation der Operation (OR 1,62, CI95 % 1,53–1,73, *p* ≤ 0,001) als signifikant für eine VWD > 7 Tage sind. Das Geschlecht, der Zugang zu Beginn der Operation und die Klassifikation nach Hansen und Stock zeigten keinen signifikanten Einfluss (Tab. 6).

**Tab. 6:** Multivariate Analyse der Krankenhausverweildauer des Registers „LapSigma“ 2013 bis 2024

	Odds Ratio (CI95 %)	*p*-Wert
*Alter*	1,03 (1,02–1,03)	< 0,001
*BMI*	1,00 (1,00–1,01)	0,031
*OP-Dauer*	1,00 (1,00–1,00)	< 0,001
*Geschlecht*	1,02 (0,96–1,02)	0,542
*ASA-Klasse*	1,35 (1,29–1,42)	< 0,001
*Indikation der Operation*	1,62 (1,53–1,73)	< 0,001
*Zugang zu Beginn der Operation*	1,09 (0,99–1,12)	0,076
*Klassifikation nach Hansen und Stock*	1,02 (0,96–1,00)	0,079

## Diskussion

Im Rahmen dieser Studie wurden die Risikofaktoren für eine verlängerte VWD nach elektiver minimalinvasiver Kolonchirurgie aus den Datensätzen der StuDoQ-Register „Kolonkarzinom“ und „LapSigma“ der Jahre 2013 bis 2024 analysiert. Die mediane VWD im „Kolonkarzinom“-Register (*n* = 17.930) lag bei 8 Tagen und „LapSigma“-Register (*n* = 26.324) bei 7 Tagen.

In der multivariaten Analyse, bezogen auf den Vergleich zwischen einer VWD ≤ 8 zu > 8 Tagen, zeigte sich beim Kolonkarzinom, dass das Alter, der BMI, die OP-Dauer, die ASA-Klasse, der Zugang bei OP-Beginn, das Operationsverfahren, die Tumorlokalisation, das pT-Stadium, der Nodalstatus und der Metastasierungsstatus einen signifikanten Einfluss auf die VWD hatten. Bei näherer Betrachtung der Odds Ratios spielen trotz Signifikanz der übrigen Werte insbesondere die ASA-Klasse mit einer Odds Ratio von 1,44 (CI95 % 1,37–1,52, *p* ≤ 0,001) und die Metastasierung mit einer Odds Ratio von 1,24 (CI95 % 1,14–1,34, *p* ≤ 0,001) eine entscheidende Rolle. In der multivariaten Analyse des „LapSigma“-Registers erwies sich ebenfalls eine Vielzahl der Variablen wie das Alter, der BMI, die OP-Dauer, die ASA-Klasse und die Indikation der Operation als signifikante Einflussfaktoren auf eine VWD über 7 Tage. Ausschlaggebend waren hier erneut v. a. die ASA-Klasse mit einer Odds Ratio von 1,35 (CI95 % 1,29–1,42, *p* < 0,001) und die Indikation der Operation 1,62 (CI95 % 1,53–1,73, *p* < 0,001). Aufgrund der hohen Fallzahl zeigte sich eine Vielzahl der untersuchten Variablen als Einflussfaktoren hinsichtlich der VWD, obwohl sie nur einen kleinen Effekt hatten.

Es konnte aufgezeigt werden, dass als wichtigster Risikofaktor für eine verlängerte VWD nach elektiver Kolonchirurgie die ASA-Klasse gilt. Das erklärt sich im Wesentlichen aus der erhöhten perioperativen Morbidität, welche mit diesem Kollektiv verbunden ist. Patientinnen und Patienten der ASA-Klasse 3 weisen mit 48 % beim Kolonkarzinom und 52 % bei der Sigmadivertikulitis eine verlängerte VWD auf.

Bei Analyse des „Kolonkarzinom“-Registers ist auffällig, dass 2024 immer noch nur 50 % der Patientinnen und Patienten mit Kolonkarzinom minimalinvasiv operiert wurden, obwohl die onkologische Gleichwertigkeit der minimalinvasiven Chirurgie (MIC) längst belegt wurde und ihre Vorteile hinsichtlich einer schnelleren Genesung ebenfalls durch hochwertige Evidenz demonstriert wurden [7, 10]. Aloia et al. zeigten in der onkologischen Leberchirurgie auf, dass Patienten nach offener Operation seltener und langsamer eine zuvor definierte weiterführende onkologische Therapie erhielten [11]. Im Rahmen dieser Analyse zeigte sich, dass insbesondere Patientinnen und Patienten mit Metastasierung ein erhöhtes Risiko für eine verlängerte VWD aufwiesen. Bei geplanter Vortherapie zur Behandlung der Metastasen sollte dieses Zeitfenster bis zur Operation des Primarius für eine Prähabilitation genutzt werden. Außerdem sollten durch einen minimalinvasiven Eingriff diese Patientinnen und Patienten schneller einer weiteren onkologischen Therapie zugeführt werden [11].

In der multivariaten Analyse des „LapSigma“-Registers zeigte sich ebenfalls die Indikation der Operation als signifikanter Prädiktor für einen verlängerten Krankenhausaufenthalt. Insbesondere die Patientinnen und Patienten mit einer elektiven Operation bei Sigmadivertikelblutung hatten mit 59 % einen verlängerten Krankenhausaufenthalt von > 7 Tagen. In dieser Kohorte spielte vermutlich bei stattgehabter oder persistierender Blutung eine Anämie eine gewisse Rolle hinsichtlich der VWD-Verlängerung. Bei Patientinnen und Patienten mit kolorektalem Karzinom und Anämie führt die Anämie zu einem schlechteren Gesamtüberleben und krankheitsfreiem Überleben [12]. Daher sollten Patientinnen und Patienten mit einer Anämie vor einem Koloneingriff präoperativ eine intravenöse Eisengabe im Kontext eines adäquaten Patient-Blood-Managements erhalten [6].

Insgesamt zeigte sich sowohl beim Kolonkarzinom als auch bei der Sigmadivertikulitis eine tendenziell abnehmende mediane und mittlere Verweildauer über die letzte Dekade. Bereits Anfang der 2000er-Jahre wurde von Kehlet et al. eine potenzielle Entlassung nach minimalinvasiver Kolonchirurgie innerhalb von 2 bis 3 Tagen propagiert. Wobei sich durch eine Entlassung nach 3 Tagen die Wiederaufnahmerate signifikant reduzierte [13].

Gemäß aktuellem DRG-Fallzahlkatalog 2025 befinden sich die meisten hier untersuchten Operationen im Bereich der DRG G02A bzw. G02B [14]. Hieraus resultieren eine untere Grenzverweildauer von 4 bis 5 Tagen, die VWD liegt bei 18,6 und 13,5 Tagen [14]. Die mittlere VWD der minimalinvasiv operierten Patienten aus den analysierten Registern lag 2024 mit 9,15 (± 13,41) Tagen beim „Kolonkarzinom“-Register und 8,08 (± 5,33) Tagen beim „LapSigma“-Register deutlich darunter. Die geplanten Mindestmengen in der onkologischen Kolonchirurgie, die schrittweise ab 2027 mit 20 Patienten pro Jahr erhöht werden sollen, wird zu einer weiteren Zentralisierung der Behandlung führen. Aus den bereits vorliegenden Daten wird deutlich, dass Zentren, die ihre Daten in das StuDoQ-Register eingeben, ihre VWD im Vergleich zu den Daten des Inek (Institut für das Entgeltsystem im Krankenhaus) halbieren konnten [14]. Eine höhere Mindestmenge der jährlichen Operationen wird zu einer weiteren Bündelung der Expertise mit einer Optimierung der hausinternen Abläufe und im Ergebnis zu einer verminderten VWD führen. Durch die damit einhergehende Reduktion der Kosten ergibt sich auch ein ökonomischer Nutzen dieses Vorgehens.

Um eine sichere Patientenversorgung, hohe chirurgische Qualität und kurze stationäre VWD erreichen zu können, bedarf es einer hohen Expertise und Fallzahl minimalinvasiver Koloneingriffe sowie eines etablierten modernen multimodalen perioperativen Behandlungspfades, der auf den aktuellen ERAS®-Leitlinien sowie der POMGAT-Leitlinie basiert. Zur optimalen Umsetzung der ERAS®/POMGAT-Ziele und somit auch kurzen VWD bedarf es eines geschulten multidisziplinären Teams sowie spezialisierter Fachkräfte/ERAS®-Nurses, die die Patienten perioperativ unterstützen [15]. Diese Risikogruppe der Patienten mit einer ASA-Klasse ≥ 3 gilt es, optimal auf den geplanten elektiven Eingriff im Sinne einer Prähabilitation vorzubereiten. Anael Barberan-Garcia et al. konnten bereits aufzeigen, dass in dem Kollektiv der Hochrisikopatienten mit einem Alter von > 70 Jahren und der ASA-Klasse 3 oder 4 durch eine 4‑wöchige Prähabilitation das Komplikationsrisiko signifikant um 50 % reduziert werden kann [16].

### Limitation

Die Studie wird limitiert durch ihr retrospektives Studiendesign. Darüber hinaus sind im StuDoQ-Register „LapSigma“ nicht alle Variablen auf dem aktuellen Stand der deutschen Leitlinie, wie z. B. die Klassifikation nach Hansen und Stock, die inzwischen durch die „Classification of Diverticular Disease“ (CCD) abgelöst wurde (S3-Leitlinie Divertikelkrankheit/Divertikulitis) [3].

## Fazit

Ein wesentlicher Risikofaktor für eine verlängerte Verweildauer nach elektiver Kolonchirurgie ist eine erhöhte ASA-Klasse (≥ 3). Darüber hinaus spielt beim Kolonkarzinom die Metastasierung eine Rolle und bei der Sigmadivertikulitis die Indikation der Operation. Patientinnen und Patienten, bei denen eine elektive Kolonresektion indiziert ist, sollten perioperativ hinsichtlich der identifizierten Risikofaktoren evaluiert werden, um drohender postoperativer Morbidität und einer Verweildauerverlängerung entgegenzuwirken [3]. Diese bereits präoperativ bekannten Risikofaktoren sollten zum Anlass genommen werden, die entsprechenden Patientinnen und Patienten in ein geeignetes Prähabilitationsprogramm einzuschließen, um dieses Kollektiv optimal auf den operativen Eingriff vorzubereiten. Insbesondere die Patienten mit einem metastasierten Kolonkarzinom profitieren von einer raschen Erholung nach der Operation, sodass eine potenziell notwendige Systemtherapie oder die Resektion von Metastasen rasch angestrebt werden kann.

## Supplementary Information

Zusätzliche Infobox 1: Teilnehmende Kliniken Kolonkarzinom

Zusätzliche Infobox 2: Teilnehmende Kliniken LapSigma

## Data Availability

Die erhobenen Datensätze können auf begründete Anfrage in anonymisierter Form bei der korrespondierenden Autorin angefordert werden. Die Daten befinden sich auf einem Datenspeicher am Universitätsklinikum Mannheim.
