# Supplementary material for: Risk factors for an extended hospital stay after elective minimally invasive colonic surgery in Germany—Data analysis of the StuDoQ register
Source: Chirurgie (Heidelb). 2026 May 26;97(9):756–64. [Article in German] doi: 10.1007/s00104-026-02510-4 (PMC13623765; doi:10.1007/s00104-026-02510-4)
Supplement: Supplementary file 1 [file 104_2026_2510_MOESM1_ESM.pdf]

Risikofaktoren für einen verlängerten Krankenhausaufenthalt nach elektiver minimalinvasiver Kolonchirurgie in Deutschland – Datenanalyse der StuDoQ-Register

M. Kaufmann (1), S. Hetjens (2), C. Klinger (3), C. Reißfelder (1), J. Hardt (1)

1 Universitätsmedizin Mannheim, Chirurgische Klinik, Mannheim, Deutschland

2 Universitätsmedizin Mannheim, Medizinische Statistik, Biomathematik und Informationsverarbeitung, Mannheim, Deutschland

3 Deutsche Gesellschaft für Allgemein- und Viszeralchirurgie. StuDoQ-Register

Teilnehmende Kliniken Kolonkarzinom

Prof. Dr. med. Mia Kim
Klinik für Allgemein-, Viszeralchirurgie und Koloproktologie, München Klinik gGmbH, Klinikum Neuperlach, München, Deutschland

Prof. Dr. med. Matthias Anthuber
Klinik für Allgemein-, Viszeral- und Transplantationschirurgie Universitätsklinikum Augsburg, Augsburg, Deutschland

Prof. Dr. med. Stefan Farkas
Klinik für Allgemein- und Visceralchirurgie, St.-Josefs-Hospital Wiesbaden GmbH, Wiesbaden, Deutschland

PD Dr. Dr. habil. Christian Mönch
Klinik für Allgemein-, Viszeral- und Transplantationschirurgie, Westpfalz-Klinikum Kaiserslautern, Kaiserslautern, Deutschland

Prof. Dr. med. Jörg-Peter Ritz
Klinik für Allgemein- und Viszeralchirurgie, Helios Kliniken Schwerin, Schwerin, Deutschland

Prof. Dr. med. Dimitrios Pantelis
Abteilung für Allgemein- und Viszeralchirurgie ,GFO Kliniken Bonn/ St. Marien-Hospital, Bonn, Deutschland

Prof. Dr. med. Stephan Coerper
Klinik für Allgemeine-, Viszeral- und Gefäßchirurgie, Krankenhaus Martha-Maria gGmbH, Nürnberg, Deutschland

Prof. Dr. med. Nico Schäfer
Klinik für Allgemein-, Visceral- und Thoraxchirurgie, Klinikum Leverkusen gGmbH, Leverkusen, Deutschland

Prof. Dr. med. Thomas Schiedeck
Klinik für Allgemein- und Viszeralchirurgie, Klinikum Ludwigsburg, Ludwigsburg, Deutschland

Prof. Dr. Christoph Reißfelder
Chirurgische Klinik, Universitätsmedizin Mannheim, Mannheim, Deutschland

Dr. med. Axel Döhrmann
Klinik für Allgemein- und Viszeralchirurgie, AMEOS Klinikum St. Clemens Oberhausen, Oberhausen, Deutschland

Lars Haeder
Allgemein- und Viszeralchirurgie, Diakovere Krankenhaus gGmbH – Henriettenstift, Hannover, Deutschland

Prof. Dr. med. Hendrik Schimmelpenning
Klinik für Allgemein-, Viszeral- und Gefäßchirurgie, Schön Klinik Neustadt, Neustadt, Deutschland

Prof. Dr. Georg A. Pistorius
Klinik für Allgemein- und Viszeralchirurgie, Sozialstiftung Bamberg, Klinik am Bruderwald, Bamberg, Deutschland

PD Dr. med. Christian Jurowich
Klinik für Allgemein-, Viszeral- und Thoraxchirurgie, InnKlinikum Altötting und Mühldorf, Altötting, Deutschland

Dr. med. Steffen Krummbein
Klinik für Allgemein-, Viszeral- und MIC, Kreiskliniken Herford-Bünde, Standort Lukas-Krankenhaus Bünde, Bünde, Deutschland

Dr. med. Ulrich Keßler
Chirurgie, Oberlausitz-Kliniken GmbH, Bautzen, Deutschland

Prof. Dr. med. Christoph-Thomas Germer
Klinik und Poliklinik für Allgemein-, Viszeral-, Transplantations-, Gefäß- und Kinderchirurgie, Universitätsklinikum Würzburg, Würzburg, Deutschland

Dr. med. Herry Helfritzsch
Klinik für Allgemein- und Viszeralchirurgie, Thüringen-Kliniken Georgius Agricola GmbH, Saalfeld, Deutschland

Prof. Dr. med. Johannes Schmidt
Chirurgische Klinik I, Krankenhaus Landshut-Achdorf, Landshut, Deutschland

Prof. Dr. med. Peter Kienle
Allgemein- und Viszeralchirurgie, Theresienkrankenhaus und Diako gGmbH, Mannheim, Deutschland

Thomas Lorenz
Chirurgische Abteilung I, St. Elisabeth-Krankenhaus Leipzig gGmbH, Leipzig, Deutschland

Dr. med. Konstantinos Zarras
Klinik für Viszeral-, Minimalinvasive und Onkologische Chirurgie, Marien Hospital, Düsseldorf, Deutschland

Dr. med. Skander Bouassida
Klinik für Koloproktologie, Vivantes-Humboldt-Klinikum Berlin, Berlin, Deutschland

Dr. med. Bernhard J. Lammers
Coloproktologie und Hernienchirurgie Rheinland Klinikum Neuss GmbH – Lukaskrankenhaus, Neuss, Deutschland

Prof. Dr. med. Johann Pratschke
Chirurgische Klinik am Campus Charité Mitte und Virchow Klinikum, Charité -Universitätsmedizin Berlin, Berlin, Deutschland

Dr. med. Alexander Buia
Chirurgie, Asklepios Klinik Langen, Langen, Deutschland

Prof. Dr. med. Anton J. Kroesen
Klinik für Allgemein-, Viszeral- und Unfallchirurgie, Krankenhaus Porz am Rhein gGmbH, Köln, Deutschland

Dr. med. Johann Strandborg
Chirurgie, Ubbo-Emmius-Klinik Aurich gGmbH, Aurich, Deutschland

Prof. Dr. Tobias Keck
Klinik für Chirugie, Universitätsklinikum Schleswig-Holstein Campus Lübeck,Lübeck, Deutschland

Prof. Dr. med. Christoph Isbert
Klinik für Allgemein-, Viszeral- und koloproktologische Chirurgie, Ev. Malie Sieveking-Krankenhaus gGmbH, Hamburg, Deutschland

Dr.(rus) Erich Keller
Abteilung für Allgemein- und Viszeralchirurgie , Krankenhaus St. Josef, Schweinfurt, Deutschland

Prof. Dr. med. Jörg Tschmelitsch
Chirurgie, Barmherzige Brüder St. Veit, St. Veit, Österreich

PD Dr. Dr. med. Uwe Johannes Roblick
Allgemein-, Viszeral- und minimalinvasive Chirurgie, Agaplesion Diakonieklinikum Hamburg gGmbH, Hamburg, Deutschland

Dr. med. Guido Hartung
Klinik für Allgemein-, Viszeral- und minimal invasive Chirurgie, Sana Klinikum Remscheid GmbH, Remscheid, Deutschland

Prof. Dr. med. Robert Rosenberg
Klinik für Allgemein-, Viszeral-, Gefäss- und Thoraxchirurgie, Kantonsspital Baselland, Liestal, Schweiz

Dr. med. Detlef Krenz
Klinik für Allgemein- und Viszeralchirurgie, Klinikum Dritter Orden München gGmbH, München, Deutschland

Prof. Dr. med. Waldemar Uhl
Klinik für Allgemein- und Viszeralchirurgie, Katholisches Klinikum Bochum gGmbH | St. Josef-Hospital, Bochum, Deutschland

Prof. Dr. med. Thorsten Vowinkel
Klinik für Allgemein-, Viszeral- und Gefäßchirurgie, RKK St. Josefskrankenhaus, Freiburg im Breisgau, Deutschland

PD Dr. med. Matthias Kraemer
Abteilung für Allgemein- und Viszeralchirurgie/Koloproktologie, St. Barbara-Klinik Hamm-Heessen GmbH, Hamm, Deutschland

PD Dr. med. Gabriele Böhm
Koloproktologie, Stiftungsklinikum Proselis gGmbH, Recklinghausen, Deutschland

Dr. med. Thorsten Jacobi
Chirurgie, Diakonissenkrankenhaus Dresden, Dresden, Deutschland

Prof. Jörg Köninger
Klinik für Allgemein-, Viszeral-, Thorax- und Transplantationschirurgie, Klinikum der Landeshauptstadt Stuttgart gKAöR – Katharinenhospital, Stuttgart, Deutschland

Dr. med. Taiseer Sayegh
Chirurgische Klinik, Marienhausklinik St. Josef Kohlhof, Neunkirchen,Deutschland

Prof. Dr. med. Daniel Vallböhmer
Klinik für Allgemein- und Viszeralchirurgie, Ev. Klinikum Niederrhein gGmbH, Duisburg, Deutschland

Prof. Dr. med. Markus Kleemann
Allgemein- und Viszeralchirurgie, Kliniken Dr. Erler gGmbH, Nürnberg, Deutschland

Dr. med. Marc Alexander Renter
Abteilung für Allgemein- und Viszeralchirurgie, Proktologie, St. Josef Krankenhaus GmbH Moers, Moers, Deutschland

Dr. med. Thies Daniels
Klinik für Allgemein-, Viszeral- und Tumorchirurgie, Albertinen-Krankenhaus, Hamburg, Deutschland

Dr. med. Muneer Deeb
Allgemein- und Visceralchirurgie, Ammerland-Klinik GmbH, Westerstede, Deutschland

Prof. Dr. med. Matthias Kemen
Allgemein- und Viszeralchirurgie, Ev. Krankenhaus Herne, Herne, Deutschland

Dr. med. Claus Schöler
Allgemein- und Viszeralchirurgie, Wertachklinik Schwabmünchen, Schwabmünchen, Deutschland

Prof. Dr. med. Jens Werner
Klinik für Allgemeine, Viszeral-, Transplantations-, Gefäß- und Thoraxchirurgie, Klinikum der Universität München, Klinikum Großhadern, München, Deutschland

PD Dr. med. Franz Georg Bader
Isar Kliniken GmbH, München,Deutschland

Dr. Volker Fackeldey
Chirurgie, Klinik Kitzinger Land , Kitzingen, Deutschland

PD Dr. med. Silke Schüle
Chirurgische Klinik, Waldkrankenhaus St. Marien, Erlangen, Deutschland

Dr. Patrick von Parpart
Allgemein- und Viszeralchirurgie, Krankenhaus Friedberg, Kliniken an der Paar, Friedberg, Deutschland

PD Dr. med. Asad Kutup
Allgemein- und Viszeralchirurgie, Asklepios Klinik Nord, Standort Heidberg, Hamburg, Deutschland

Dr. med. Carmen Klein
Klinik für Allgemein- und Viszeralchirurgie, Westpfalz-Klinikum GmbH Standort III, Kirchheimbolanden, Deutschland

Prof. Dr. med. Marco Niedergethmann
Klinik für Allgemein- und Viszeralchirurgie, Alfried-Krupp-Krankenhaus, Essen, Deutschland

Prof. Dr. med. Torsten Wilhelm
Allgemein- und Viszeralchirurgie, GRN-Klinik Weinheim, Weinheim, Deutschland

Prof. Dr. med. Peter Markus
Allgemein-, Viszeral- und Unfallchirurgie, Elisabeth-Krankenhaus Essen GmbH, Essen, Deutschland

Dr. med. Claus Schneider
Allgemein-, Viszeral- und Gefäßchirurgie, Marienhausklinikum St. Elisabeth, Neuwied, Deutschland

Dr. med. Ulrike Soetje
Abteilung für Koloproktologie, St. Josefs-Hospital Wiesbaden GmbH, Wiesbaden, Deutschland

PD Dr. med. habil. Christoph Strey
Abteilung für Allgemein- und Viszeralchirurgie, DRK-Krankenhaus Clementinenhaus, Hannover, Deutschland

Dr. med. Jochen Klinnert
Abteilung für Allgemein- und Viszeralchirurgie , Asklepios Klinik Parchim, Parchim, Deutschland

Prof. Dr. med. Christian Peiper
Abteilung Allgemein und Visceralchirurgie, EVK Hamm Evangelisches Krankenhaus, Hamm, Deutschland

Dr. med. Bernhard Drummer
Allgemeinchirurgische Abteilung, Klinikum Forchheim - Fränkische Schweiz gGmbH, Forchheim, Deutschland

Dr. Alexander Janzen
Klinik für Chirurgie, Christliches Klinikum Unna Mitte, Unna, Deutschland

Dr. med. Toralf Wolkersdörfer
Allgemein- und Viszeralchirurgie, Ilm-Kreis-Kliniken, Arnstadt, Deutschland

Dr. med. Hans-Peter Wüllenweber
Klinik für Allgemein- und Viszeralchirurgie, Marienhospital Aachen, Aachen, Deutschland

Prof. Dr. med. Jörg C. Kalff
Abteilung für Viszerale/kolorektale Chirurgie und Proktologie, Universitätsklinikum Bonn, Bonn, Deutschland

Prof. Dr. med. Christiane Bruns
Klinik und Poliklinik für Allgemein-, Viszeral- und Tumor- und Transplantationschirurgie, Uniklinik Köln, Köln, Deutschland

Dr. med. Alexander Meyer
Klinik für Allgemein-, Viszeral- und Gefäßchirurgie und Koloproktologie, Johanniter Krankenhaus Rheinhausen, Duisburg, Deutschland

Dr. med. Tina Maghsoudi
Klinik für Allgemein- und Viszeralchirurgie, Schön Klinik Hamburg Eilbek, Hamburg, Deutschland

Dr. med. Frank Klammer
Allgemein-, Viszeral- und Thoraxchirurgie, St. Franziskus-Hospital, Ahlen, Deutschland

Dr. med. Uwe Werner
Mühlenkreiskliniken (AöR) - Krankenhaus Lübbecke, Lübbecke, Deutschland

PD Dr. med. Merten Hommann
Allgemeine Chirurgie, Viszeralchirurgie, Zentralklinik Bad Berka, Bad Berka, Deutschland

PD Dr. med. Christian Kuntz
Allgemein- u. Visceralchirugie/Proktologie, Kreiskrankenhaus Weilburg gGmbH, Weilburg, Deutschland

Dr. med. Michael Zaczek
Klinik für Allgemeine und Spezielle Viszeralchirurgie, Philippusstift Essen-Borbeck, Essen, Deutschland

Dr. med. univ. Florian Stadler
Allgemein- und Visceralchirurgie, Proktologie, Sana Kliniken des Landkreises Cham, Cham, Deutschland

Dr. med. Jens Jonescheit
Allgemein- und Viszeralchirurgie, Heilig-Geist-Hospital, Bensheim, Deutschland

Dr. med. Sigurd Hanke
Chirurgische Klinik, KKH Delitzsch GmbH, Klinik Eilenburg, Delitzsch, Deutschland

Prof. Dr. med. Jörg Glatzle
Allgemein- und Viszeralchirurgie, Klinikum Konstanz, Konstanz, Deutschland

Dr. med. Ralf Uhlig
Klinik für Chirurgie, St.-Josef-Krankenhaus, Potsdam, Deutschland

PD Dr. med. Johannes Heimbucher
Chirurgische Klinik, Marienkrankenhaus Kassel gGmbH, Kassel, Deutschland

Dr. med. Colin M. Krüger
Chirurgie, Immanuel Klinik Rüdersdorf, Rüdersdorf, Deutschland

Alexander Friedemann
Klinik für Allgemein- und Viszeralchirurgie, Rotes Kreuz Krankenhaus Bremen gGmbH, Bremen, Deutschland

Dr. med. Lutz Hausser
Allgemein- und Viszeralchirurgie, Klinikverbund Allgäu, Klinik Immenstadt, Immenstadt, Deutschland

Prof. Dr. med. Christoph Heidenhain
Klinik für Allgemein- und Viszeralchirurgie, Agaplesion Markus Krankenhaus, Frankfurt, Deutschland

Prof. Dr. med. Olaf Horstmann
Allgemein-, Viszeral- und Endokrine Chirurgie,Städtische Kliniken Mönchengladbach, Mönchengladbach, Deutschland

Dr. med. Christian Hessler
Allgemein-, und Viszeralchirurgie, Proktologie, MIC, Heilig-Geist-Hospital, Bingen, Deutschland

Dr. med. Lars Brinkmann
Allgemein-, Viszeral- und Minimal invasive Chirurgie, St. Vinzenz Krankenhaus Hanau, Hanau, Deutschland

Dr. med. Harald Tigges
Allgemein-, Viszeral- und Gefäßchirurgie, Klinikum Landsberg am Lech, Landsberg am Lech, Deutschland

Dr. med. Alexander Terzic
Klinik für Allgemein-, Visceral-, und Gefäßchirurgie, Krankenhaus Johanneum, Wildeshausen, Deutschland

Dr. med. Steffen Lancee
Allgemein-, Visceral- und Gefäßchirurgie, Kreiskrankenhaus Vogelsbergkreis GmbH, Alsfeld, Deutschland

Dr. med. Christoph Reuter
Allgemein- und Viszeralchirurgie, Proktologie, Christliches Krankenhaus Quakenbrück, Quakenbrück, Deutschland

Prof. Dr. med. Metin Senkal
Allgemein- und Viszeralchirurgie, Marien-Hospital Wittem, Witten, Deutschland

Dr. med. Christoph Ulmer
Allgemein-, Viszeral- und Gefäßchirurgie, Rems-Murr-Klinik Schorndorf, Schorndorf, Deutschland

Dr. med. Matthias Berger
Allgemein-, Viszeralchirurgie und Proktologie, Diakoniekrankenhaus Chemnitzer Land, Hartmannsdorf, Deutschland

Dr. med. Ralf Weise
Klinik für Allgemein- und Visceralchirurgie, St.-Marien-Hospital, Friesoythe, Deutschland

Dr. med. Dirk Meisel
Allgemein-, Viszeral- und Kinderchirurgie, Kreiskrankenhaus Freiberg gGmbH, Freiberg, Deutschland

Prof. Dr. med. Daniel W. Kauff
Klinik für Allgemein-, Viszeral- und Gefäßchirurgie mit Unfallchirurgie Nagold, Klinikverbund Südwest GmbH - Kliniken Nagold, Nagold, Deutschland

PD Dr. med. René Hennig
Allgemein-, Viszeral- und Gefäßchirurgie, Krankenhäuser Landkreis Freudenstadt, Freudenstadt, Deutschland

Dr. Henryk Thielemann
Chirurgie, BG Klinikum Unfallkrankenhaus Berlin gGmbH, Berlin, Deutschland

Dr. med. Christine Wurst
Allgemein- und Viszeralchirurgie, St. Anna-Krankenhaus, Sulzbach-Rosenberg, Deutschland

Prof. Dr. med. Robert Grützmann
Chirurgische Klinik, Universitätsklinikum Erlangen, Erlangen, Deutschland

Dr. med. Sigurd Hanke
Abteilung für Allgemein- und Viszeralchirurgie, KKH Delitzsch GmbH, Klinik Delitzsch, Delitzsch, Deutschland

Prof. Dr. med. Hans-Bernd Reith
Allgemein- und Viszeralchirurgie, Agaplesion Diakonie Kliniken Kassel gGmbH, Kassel, Deutschland

Dr. med. Mark Banysch
Klinik für Allgemein- und Viszeralchirurgie, St. Bernhard-Hospital Kamp-Lintfort GmbH, Kamp-Lintfort, Deutschland

Dr. med. Markus Wittschier
Allgemein-, Viszeral- und Gefäßchirurgie, St. Barbara-Hospital, Gladbeck, Deutschland

Dr. med. Verena Grünewald
Allgemein- und Visceralchirurgie, St. Elisabeth-Krankenhaus Geilenkirchen gGmbH, Geilenkirchen, Deutschland

PD Dr. med. Burkhard Stoffels
Klinik für Allg.-, Viszeral- u. Unfallchirurgie, Heilig Geist-Krankenhaus GmbH, Köln, Deutschland

Dr. med. Markus Wittschier
Klinik für Allgemein- und Viszeralchirurgie, Sankt Marien-Hospital Buer GmbH, Gelsenkirchen, Deutschland

Dr. med. Roland Hutzschenreuter
Allgemein-und Viszeralchirurgie, Kath. Kliniken Oberberg gGmbH Engelskirchen, Engelskirchen, Deutschland

Tim Wessels
Allgemein-, Gefäß- und Viszeralchirurgie, Ludgerus-Kliniken Münster GmbH – Clemenshospital, Münster, Deutschland

Sorbin Serban
Allgemein-und Visceralchirurgie, St. Josefs Krankenhaus Balserische Stiftung, Gießen, Deutschland

Prof. Dr. med. Moritz Schmelzle
Klinik für Allgemein-, Viszeral- und Transplantationschirurgie,Med. Hochschule Hannover, Hannover, Deutschland

Kai Etzkorn
Allgemein- und Viszeralchirurgie, St. Marien Krankenhaus GmbH, Ratingen, Deutschland

PD Dr. med. Claudia Rudroff
Allgemein- und Viszeralchirurgie, Evangelisches Krankenhaus Köln-Weyertal gGmbH, Köln, Deutschland

Prof. Dr. med. Andreas Rink
Allgemein-, Viszeral- und Transplantationschirurgie, Universitätsklinikum Essen, Essen, Deutschland

Dr. med. Burkhard Thiel
Chirurgische Klinik, Knappschaft Kliniken Lünen, Lünen, Deutschland

Matthias Blase
Klinik für Allgemein- und Viszeralchirurgie, EvK Witten gGmbH, Witten, Deutschland

PD Dr. med. Matthias Goos
Allgemein- und Viszeralchirurgie, Helios Klinikum Müllheim, Müllheim, Deutschland

Prof. Dr. Katharina Beyer
Klinik für Allgemein-, Viszeral- und Gefäßchirurgie, Charité - Universitätsmedizin Berlin, Campus Benjamin Franklin, Berlin, Deutschland

Dr. med. Daniel Ditterich
Allgemein-, Viszeral- und Gefäßchirurgie, Klinikum Neustadt a. d. Aisch, Neustadt a. d. Aisch, Deutschland

Prof. Dr. med. Tung Yu Tsui
Klinik für Allgemein- Viszeral- und Thoraxchirurgie, Asklepios Harzkliniken GmbH, Goslar, Deutschland

Dr. med. Thomas Schäller
Klinik für Allgemein- und Viszeralchirurgie, Erzgebirgsklinikum gGmbH, Haus Annaberg, Annaberg-Buchholz, Deutschland

Prof. Dr. med. Pompiliu Piso
Klinik für Allgemein- und Viszeralchirurgie, Krankenhaus Barmherzige Brüder Regensburg, Regensburg, Deutschland

PD Dr. med. Michael Knoop
Klinik für Allgemein- und Viszeralchirurgie, Klinikum Frankfurt/Oder, Frankfurt/Oder, Deutschland

Dr. med. Gregor Jung
Klinik für Allgemein- und Viszeralchirurgie, Kreiskrankenhaus Bergstraße gemeinnützige GmbH, Heppenheim, Deutschland

PD Dr. Florian Herrle
Allgemein- und Viszeralchirurgie, RoMed Klinik am Chiemsee , Prien, Deutschland

Dr. med. Arkadiy Radzikhovskiy
Klinik für Allgemein- & Viszeralchirurgie, Kreiskrankenhaus Frankenberg gGmbH, Frankenberg, Deutschland

Prof. Dr. med. Dr. habil. Thomas Carus
Abteilung für Allgemein- und Viszeralchirurgie,Klinik Bassum, BassumDeutschland

Dr. med.Hans-Jürgen Hain
Klinik für Allgemeinchirurgie, Kreisklinik Groß-Umstadt, Groß-Umstadt, Deutschland

Dr. med. Frank Starp
Klinik für Allgemein- und Viszeralchirurgie, Delme Klinikum Delmenhorst, Delmenhorst, Deutschland
