# Supplementary material for: Risk factors for an extended hospital stay after elective minimally invasive colonic surgery in Germany—Data analysis of the StuDoQ register
Source: Chirurgie (Heidelb). 2026 May 26;97(9):756–64. [Article in German] doi: 10.1007/s00104-026-02510-4 (PMC13623765; doi:10.1007/s00104-026-02510-4)
Supplement: Supplementary file 2 [file 104_2026_2510_MOESM2_ESM.pdf]

Risikofaktoren für einen verlängerten Krankenhausaufenthalt nach elektiver minimalinvasiver Kolonchirurgie in Deutschland – Datenanalyse der StuDoQ-Register

M. Kaufmann (1), S. Hetjens (2), C. Klinger (3), C. Reißfelder (1), J. Hardt (1)

1 Universitätsmedizin Mannheim, Chirurgische Klinik, Mannheim, Deutschland
2 Universitätsmedizin Mannheim, Medizinische Statistik, Biomathematik und Informationsverarbeitung, Mannheim, Deutschland
3 Deutsche Gesellschaft für Allgemein- und Viszeralchirurgie. StuDoQ-Register

Teilnehmende Kliniken LapSigma

PD Dr. med. Matthias Kraemer
Abteilung für Allgemein- und Viszeralchirurgie/Koloproktologie, St. Barbara-Klinik Hamm-Heessen GmbH, Hamm, Deutschland

Dr. med. Klaus Kraft
Allgemein- und Viszeralchirurgie, medius Klinik Nürtingen, Nürtingen, Deutschland

PD Dr. med. habil. Christoph Strey
Abteilung für Allgemein- und Viszeralchirurgie, DRK-Krankenhaus Clementinenhaus, Hannover, Deutschland

Prof. Dr. med. Dimitrios Pantelis
Abteilung für Allgemein- und Viszeralchirurgie, GFO Kliniken Bonn/ St. Marien-Hospital, Bonn, Deutschland

Prof. Dr. med. Stefan Farkas
Klinik für Allgemein- und Visceralchirurgie, St.-Josefs-Hospital Wiesbaden GmbH, Wiesbaden, Deutschland

Prof. Dr. med. Andreas Türler
Allgemein- und Viszeralchirurgie, Johanniter-Krankenhaus Bonn, Bonn, Deutschland

Dr. med. Hans-Peter Wüllenweber
Klinik für Allgemein- und Viszeralchirurgie, Marienhospital Aachen, Aachen, Deutschland

Prof. Dr. med. Christoph Jacobi
Chirurgie, Dreifaltigkeits-Krankenhaus Wesseling, Wesseling, Deutschland

Prof. Dr. med. Christian Klink
Klinik für Allgemein- und Viszeralchirurgie, Diakonissen-Stiftungs-Krankenhaus Speyer, Speyer, Deutschland

Dr. med. Mirjam Heidmann
Chirurgie, Krankenhaus Salem, Heidelberg, Deutschland

Dr. med. Barbara Kraft
Klinik für Allgemein- und Viszeralchirurgie, Diakonie-Klinikum Stuttgart, Stuttgart, Deutschland

PD Dr. med. Benno Mann
Allgemein- und Viszeralchirurgie, Augusta Krankenanstalt, Bochum, Deutschland

Prof. Dr. med. Matthias Kemen
Allgemein- und Viszeralchirurgie, Ev. Krankenhaus Herne, Herne, Deutschland

Prof. Dr. med. Matthias Pross
Klinik für Allgemein-, Viszeral- und Minimalinvasive Chirurgie, DRK-Kliniken Berlin-Köpenick, Berlin Deutschland

Dr. med. Christos Zigouris
Klinik für Allgemein-, Viszeral- und Gefäßchirurgie, Klinikum Idar-Oberstein GmbH, Idar-Oberstein, Deutschland

Prof. Dr. med. Robert Rosenberg
Klinik für Allgemein-, Viszeral-, Gefäss- und Thoraxchirurgie, Kantonsspital Baselland, Liestal, Schweiz

Dr. med. Claus Schöler
Allgemein- und Viszeralchirurgie, Wertachklinik Schwabmünchen, Schwabmünchen, Deutschland

Prof. Dr. med. Marco Siech
Chirurgie I, Ostalb-Klinikum Aalen, Aalen, Deutschland

Oberstarzt Prof. Dr. med. Robert Schwab
Klinik für Allgemein-, Viszeral- und Thoraxchirurgie, Bundeswehrzentralkrankenhaus Koblenz, Koblenz, Deutschland

Dr. med. Detlef Krenz
Klinik für Allgemein- und Viszeralchirurgie, Klinikum Dritter Orden München gGmbH, München, Deutschland

Prof. Dr. med. Jens Werner
Klinik für Allgemeine, Viszeral-, Transplantations-, Gefäß- und Thoraxchirurgie, Klinikum der Universität München, Klinikum Großhadern, München, Deutschland

Dr. med. Lu Cai
Chirurgische Abteilung, Rotkreuzklinikum München gGmbH, München, Deutschland

Dr. med. Konstantinos Zarras
Klinik für Viszeral-, Minimalinvasive und Onkologische Chirurgie, Marien Hospital, Düsseldorf, Deutschland

Prof. Dr. med. Markus Heiss
Klinik für Viszeral-, Gefäß- und Transplantationschirurgie, Kliniken der Stadt Köln gGmbH, Krankenhaus Merheim, Köln, Deutschland

Prof. Dr. med. Johannes Schmidt
Chirurgische Klinik I, Krankenhaus Landshut-Achdorf, Landshut, Deutschland

Prof. Dr. med. Dieter Birk
Klinik für Allgemein- und Viszeralchirurgie, RKH Klinik Bietigheim – Vaihingen, Bietigheim-Bissingen, Deutschland

PD Dr. med. Franz Georg Bader
Isar Kliniken GmbH, München, Deutschland

Dr. med. Elmar Kleimann
Klinik für Allgemein- und Visceralchirurgie, St. Franziskus-Hospital Köln, Köln,Deutschland

Prof. Dr. med. Erik Schlöricke
Klinik für Viszeral-, Thorax- und Gefäßchirurgie, Westküstenkliniken Brunsbüttel und Heide gGmbH, Heide, Deutschland

Dr. med. Mark Banysch
Klinik für Allgemein- und Viszeralchirurgie, St. Bernhard-Hospital Kamp-Lintfort GmbH, Kamp-Lintfort, Deutschland

Dr. med. Colin M. Krüger
Chirurgie, Immanuel Klinik Rüdersdorf, Rüdersdorf, Deutschland

Prof. Dr. med. Matthias Behrend
Viszeral-, Thorax- und Gefäßchirurgie, Donau-Isar-Klinikum Deggendorf, Deggendorf, Deutschland

Prof. Dr. med. Maximilian Schmeding
Chirurgie, Klinikum Dortmund gGmbH, Dortmund, Deutschland

PD Dr. med. Kay Kohlhaw
Allgemein-, Viszeral-, MIC- und Gefäßchirurgie, Sana Kliniken Leipziger Land GmbH, Borna, Deutschland

Dr. med. Uwe Widmaier
Abteilung für Allgemein-, Viszeral- und minimal invasive Chirurgie, Kreisklinik Günzburg , Günzburg, Deutschland

Prof. Dr. med. Ulrich Bolder
Klinik für Allgemein- und Viszeralchriurgie, St. Johannes-Hospital, Dortmund, Deutschland

Prof. Dr. med. Frank Alexander Granderath
Klinik für Allgemein- und Viszeralchirurgie, Krankenhaus Neuwerk, Mönchengladbach, Deutschland

Prof. Dr. med. Stefan Benz
Klinik für Allgemein-, Viszeral-, Thorax- und Kinderchirurgie, Kreiskliniken Böblingen gGmbH, Böblingen, Deutschland

Dr. med. Matthias Hassenpflug
Klinik für Allgemein- und Viszeralchirurgie, GRN-Klinik Sinsheim , Sinsheim, Deutschland

Prof. Dr. med. Bettina Rau
Chirurgische Klinik, Klinikum Neumarkt, Neumarkt, Deutschland

PD Dr. med. Nikos Emmanouilidis
Abteilung für Chirurgie, St. Elisabeth Hospital Gütersloh, Gütersloh, Deutschland

Prof. Dr. med. Waldemar Uhl
Klinik für Allgemein- und Viszeralchirurgie, Katholisches Klinikum Bochum gGmbH | St. Josef-Hospital, Bochum, Deutschland

PD Dr. med. Claudia Rudroff
Allgemein- und Viszeralchirurgie, Evangelisches Krankenhaus Köln-Weyertal gGmbH, Köln, Deutschland

Dr. med. Gerald Prechtl
Allgemein- und Viszeralchirurgie, Klinik Hallerwiese, Nürnberg, Deutschland

Dr. med. Friedrich Krings
Chirurgische Klinik I, Christophorus Kliniken, Coesfeld, Deutschland

PD Dr. Dr. med. Uwe Johannes
Roblick Allgemein-, Viszeral- und minimalinvasive Chirurgie, Agaplesion Diakonieklinikum Hamburg gGmbH, Hamburg, Deutschland

Prof. Dr. med. Johann Pratschke
Chirurgische Klinik am Campus Charité Mitte und Virchow Klinikum, Charité - Universitätsmedizin Berlin, Berlin, Deutschland

Dr. med. Thorsten Jacobi
Chirurgie, Diakonissenkrankenhaus Dresden, Dresden, Deutschland

Dr. med. Bernhard Drummer
Allgemeinchirurgische Abteilung, Klinikum Forchheim - Fränkische Schweiz gGmbH, Forchheim, Deutschland

Dr. med. Tina Maghsoudi
Klinik für Allgemein- und Viszeralchirurgie, Schön Klinik Hamburg Eilbek , Hamburg, Deutschland

Prof. Dr. med. Martin Angele
Abtlg. für Allgemein-, Viszeral-, Thorax- und endokrine Chirurgie, Klinikum Garmisch-Partenkirchen, Garmisch-Partenkirchen, Deutschland

Dr. med. Alexander Buia
Chirurgie, Asklepios Klinik Langen, Langen, Deutschland

Dipl. med. Britt Hinneburg
Klinik für Allgemein- und Viszeralchirurgie, Havelland Kliniken GmbH, Nauen, Deutschland

Dr. med. Marc Niewiera
Allgemein-, Viszeral- und Minimalinvasive Chirurgiem Eifelklinik St. Brigida GmbH & Co. KG, Simmerath, Deutschland

MUDr. med. David Czinner
Allgemein-, Viszeral- und Thorxchirurgie, Klinikum St. Marien, Amberg, Deutschland

Dr. Volker Fackeldey
Chirurgie, Klinik Kitzinger Land, Kitzingen, Deutschland

Prof. Dr. med. Anton J. Kroesen
Klinik für Allgemein-, Viszeral- und Unfallchirurgie, Krankenhaus Porz am Rhein gGmbH, Köln, Deutschland

Michael Zachert
Allgemein-, Viszeralchirurgie, Klinikum des Landkreises Bamberg, Burgebrach, Deutschland

Prof. Dr. med. Kai Nowak
Allgemein-, Gefäß- und Thoraxchirurgie RoMed Klinikum Rosenheim, Rosenheim, Deutschland

Prof. Dr. med. Florian Löhe
Chirurgische Klinik I, Klinikum Landshut - AdöR der Stadt Landshut, Landshut, Deutschland

Dr. med. Harald Tigges
Allgemein-, Viszeral- und Gefäßchirurgie, Klinikum Landsberg am Lech, Landsberg am Lech, Deutschland

Prof. Dr. med. Thorsten Vowinkel
Klinik für Allgemein-, Viszeral- und Gefäßchirurgie, RKK St. Josefskrankenhaus, Freiburg im Breisgau, Deutschland

PD Dr. med. Daniel Schubert
Klinik für Allgemein-, Viszeral- und Tumorchirurgie, Krankenhaus St. Elisabeth und St. Barbara (Saale) GmbH, Halle, Deutschland

Dr. med. Nurettin Albayrak
Klinik für Allgemein- und Viszeralchirurgie, St. Anna-Hospital, Herne, Deutschland

Prof. Dr. med. Marco Niedergethmann
Klinik für Allgemein- und Viszeralchirurgie, Alfried Krupp Krankenhaus, Essen, Deutschland

Dr. med. Steffen Krummbein
Klinik für Allgemein-, Viszeral- und MIC Kreiskliniken Herford-Bünde, Standort Lukas-Krankenhaus Bünde, Bünde, Deutschland

Prof. Dr. Georg A. Pistorius
Klinik für Allgemein- und Viszeralchirurgie, Sozialstiftung Bamberg, Klinik am Bruderwald, Bamberg, Deutschland

Prof. Dr. med. Torsten Wilhelm
Allgemein- und Viszeralchirurgie, GRN-Klinik Weinheim, Weinheim, Deutschland

Prof. Dr. med. Marty Zdichavsky
Allgemein-, Viszeral-, Thorax- und Unfallchirurgie, chirurgische Orthopädie Abteilung F Filderklinik gGmbH, Filderstadt, Deutschland

Dr. med. Claus Schneider
Allgemein-, Viszeral- und Gefäßchirurgie, Marienhausklinikum St. Elisabeth, Neuwied, Deutschland

Dr. med. Uwe Werner
Mühlenkreiskliniken (AöR) - Krankenhaus Lübbecke, Lübbecke, Deutschland

Prof. Dr. med. Christian Peiper
Abteilung Allgemein und Visceralchirurgie, EVK Hamm Evangelisches Krankenhaus, Hamm, Deutschland

PD Dr. med. Johannes Heimbucher
Chirurgische Klinik, Marienkrankenhaus Kassel gGmbH, Kassel, Deutschland#

Sorbin Serban
Allgemein-und Visceralchirurgie, St. Josefs Krankenhaus Balserische Stiftung, Gießen, Deutschland

Prof. Dr. med. Marco Niedergethmann
Klinik für Allgemein- und Viszeralchirurgie, Alfried Krupp Krankenhaus Essen Steele, Essen – Steele, Deutschland

Dr. med. Roland Hutzschenreuter
Allgemein-und Viszeralchirurgie, Kath. Kliniken Oberberg gGmbH Engelskirchen , Engelskirchen, Deutschland

Dr. med. Martin Laut
Klinik für Allgemein-, Viszeral- und Gefäßchirurgie, Ortenau-Klinikum, Offenburg, Deutschland

Dr. med. Norbert Hennes
Allgemein, Viszeral- und Minimal-Invasive Chirurgie, Helios Klinikum Duisburg, Duisburg, Deutschland

Dr. med. Burghard Jenert
Klinik für Allgemein-, Viszeral- und MIC Landkreis Mittweida Krankenhaus gGmbH, Mittweida, Deutschland

Prof. Dr. med. Markus Kleemann
Allgemein- und Viszeralchirurgie, Kliniken Dr. Erler gGmbH, Nürnberg, Deutschland

Prof. Dr. med. Lars Fischer
Allgemein- und Viszeralchirurgie, Klinikum Mittelbaden Baden-Baden | Klinik Balg, Baden-Baden, Deutschland

Dr. med. Christoph Ulmer
Allgemein-, Viszeral- und Gefäßchirurgie, Rems-Murr-Klinik Schorndorf, Schorndorf, Deutschland

Prof. Dr. med. Jörg Glatzle
Allgemein- und Viszeralchirurgie, Klinikum Konstanz, Konstanz, Deutschland

Prof. Dr. med. Jörg Theisen
Allgemein-, Viszeral- und Thoraxchirurgie, Klinikum Landkreis Erding, Erding, Deutschland

Dr. med. Frank Klammer
Allgemein-, Viszeral- und Thoraxchirurgie, St. Franziskus-Hospital, Ahlen, Deutschland

Dr. med. Christine Wurst
Allgemein- und Viszeralchirurgie, St. Anna-Krankenhaus, Sulzbach-Rosenberg, Deutschland

Dr. med. Markus Wittschier
Allgemein-, Viszeral- und Gefäßchirurgie, St. Barbara-Hospital, Gladbeck, Deutschland

Dr. med. univ. Florian Stadler
Allgemein- und Visceralchirurgie, Proktologie, Sana Kliniken des Landkreises Cham, Cham, Deutschland

Dr. med. Klaus Kraft
Klinik für Allgemein- und Viszeralchirurgie, medius Klinik Ostfildern-Ruit, Ostfildern, Deutschland

Dr. med. Michael Kindler
Chirurgische Klinik, Helios Weißeritztal-Kliniken GmbH, Freital, Deutschland

Prof. Dr. med. Andreas Raffel
Klinik für Allgemein-, Viszeral- und Endokrine Chirurgie Marienhospital Gelsenkirchen GmbH, Gelsenkirchen, Deutschland

Dr. Alexander Janzen
Klinik für Chirurgie, Christliches Klinikum Unna Mitte, Unna, Deutschland

Dr. med. Fabian A. Helfritz
Klinik für Allgemein- und Viszeralchirurgie, Bürgerhospital FFM, Frankfurt am Main, Deutschland

Dr. med. Markus Wagner
Chirurgie, Klinikum Seefeld, Seefeld, Deutschland

PD Dr. med. René Hennig
Allgemein-, Viszeral- und Gefäßchirurgie, Krankenhäuser Landkreis Freudenstadt, Freudenstadt, Deutschland

Dr. med. Swantje Wienand
Allgemein-, Viszeral- und Unfallchirurgie, Klinikum Links der Weser, Bremen, Deutschland

Dr. med. Markus Wittschier
Klinik für Allgemein- und Viszeralchirurgie, Sankt Marien-Hospital Buer GmbH, Gelsenkirchen, Deutschland

Prof. Dr. med. Jörg C. Kalff
Abteilung für Viszerale/kolorektale Chirurgie und Proktologie, Universitätsklinikum Bonn, Bonn, Deutschland

Prof. Dr. med. Claus Eisenberger
Klinik für Chirurgie, Krankenhaus Holweide, Köln, Deutschland

Dr. med. Golo Brodik
Allgemein- und Viszeralchirurgie, Marienkrankenhaus Soest, Soest, Deutschland

Dr. med. Alexander Terzic
Klinik für Allgemein-, Visceral-, und Gefäßchirurgie, Krankenhaus Johanneum, Wildeshausen, Deutschland

Prof. Dr. med. Daniel W. Kauff
Klinik für Allgemein-, Viszeral- und Gefäßchirurgie mit Unfallchirurgie Nagold, Klinikverbund Südwest GmbH - Kliniken Nagold , Nagold, Deutschland

Prof. Dr. med. Daniel Seehofer
Klinik und Poliklinik für Viszeral-, Transplantations-, Thorax- und Gefäßchirurgie Universitätsklinikum Leipzig AöR, Leipzig, Deutschland

Dr. med., Michael, Zaczek, Klinik für Allgemeine und Spezielle Viszeralchirurgie Philippusstift Essen-Borbeck, Essen, Deutschland

Dr. med. Gunnar Proff
Abteilung für Allgemein-, Viszeral- und Unfallchirurgie Krankenhaus St. Marienwörth, Bad Kreuznach, Deutschland

Dr. med. Christian Plötz
Klinik für Allgmein-, Viszeral- und Minimalinvasive Chirurgie, Klinikum Bad Hersfeld GmbH, Bad Hersfeld, Deutschland

Dr. med. Dirk Meisel
Allgemein-, Viszeral- und Kinderchirurgie, Kreiskrankenhaus Freiberg gGmbH, Freiberg, Deutschland

PD Dr. med. Matthias Goos
Allgemein- und Viszeralchirurgie, Helios Klinikum Müllheim, Müllheim, Deutschland

PD Dr. med. Merten Hommann
Allgemeine Chirurgie, Viszeralchirurgie Zentralklinik Bad Berka, Bad Berka, Deutschland

Prof. Dr. Martin Stockmann
Klinik für Allgemein-, Viszeral- und Gefäßchirurgie, EvK Paul-Gerhardt-Stift, Lutherstadt Wittenberg, Deutschland

Alexander Friedemann
Klinik für Allgemein- und Viszeralchirurgie, Rotes Kreuz Krankenhaus Bremen gGmbH, Bremen, Deutschland

Dr. med. Jörg Celesnik
Allgemein- und Viszeralchirurgie, Knappschafts-Krankenhaus Bottrop, Bottrop, Deutschland

Dr. med. Robert Koburg
Allgemein- und Viszeralchirurgie, Regiomed Klinikum Hildburghausen, Hildburghausen, Deutschland

Dr. med. Muharrem Saklak
Klinik für Allgemein- Viszeral- und minimalinvasive Chirurgie, DRK Kliniken Berlin-Mitte, Berlin, Deutschland

Thomas Lorenz
Chirurgische Abteilung I, St. Elisabeth-Krankenhaus Leipzig gGmbH, Leipzig, Deutschland

Dr. med. Konstantinos Lioupis
Abteilung für Chirurgie, Städtisches Krankenhaus Heinsberg GmbH, Heinsberg, Deutschland

Dr. med. Steffen Lancee
Allgemein-, Visceral- und Gefäßchirurgie, Kreiskrankenhaus Vogelsbergkreis GmbH, Alsfeld, Deutschland

Dr. med. Jens Burghardt
Allgemein- und Viszeralchirurgie, Vivantes Klinikum Kaulsdorf, Berlin, Deutschland

PD Dr. med. Henning Mothes
Klinik für Allgemein-, Viszeral- und Gefäßchirurgie, Sophien- und Hufeland-Klinikum Weimar gGmbH, Weimar, Deutschland

Prof. Dr. med. Maximilian Bockhorn
Klinik für Allgemein- und Viszeralchirurgie, Klinikum Oldenburg, Oldenburg, Deutschland

Dr. med. Carsten Bismarck
Klinik für Allgemein-, Viszeral- und Adipositaschirurgie, Hospital zum Heiligen Geist, Fritzlar, Deutschland

Dr. med. Min-Seop Son
Allgemein- und Viszeralchirurgie, WolfartKlinik Gräfelfing, Gräfelfing, Deutschland

Dr. med. Viorel Fetcu Chirurgische
Klinik - Allgemein- und Viszeralchirurgie St. Martinus-Hospital, Olpe, Deutschland

Kai Etzkorn
Allgemein- und Viszeralchirurgie, St. Marien Krankenhaus GmbH, Ratingen, Deutschland

Dr. med. Ralf Weise
Klinik für Allgemein- und Visceralchirurgie, St.-Marien-Hospital, Friesoythe, Deutschland

Dr. med. Burkhard Thiel
Chirurgische Klinik, Knappschaft Kliniken Lünen, Lünen, Deutschland

PD Dr. med. Markus Ghadimi
Klinik für Allgemein- und Viszeralchirurgie, Klinikum Stadt Soest , Soest, Deutschland

Matthias Blase
Klinik für Allgemein- und Viszeralchirurgie, EvK Witten gGmbH, Witten, Deutschland

Dr. med. Philipp Kaudel
Allgemein-, Visceral- und spezielle Visceralchirurgie, Onkologische Chirurgie, Krankenhaus Ludmillenstift, Meppen, Deutschland

Dr. med. Daniel Ditterich
Allgemein-, Viszeral- und Gefäßchirurgie, Klinikum Neustadt a. d. Aisch, Neustadt a. d. Aisch, Deutschland

Britt Hinneburg
Klinik für Allgemein- und Viszeralchirurgie, Havelland Kliniken GmbH, Rathenow, Deutschland

Prof. Dr. med. Jens Standop
Chirurgie, DRK Krankenhaus Neuwied, Neuwied, Deutschland

Prof. Dr. med. Dr. habil. Thomas Carus
Abteilung für Allgemein- und Viszeralchirurgie, Klinik Bassum, Bassum, Deutschland

Dr. med. Stefan Bollmann
Allgemein- und Viszeralchirurgie, Helios-Klinik Attendorn, Attendorn, Deutschland

Dr. med. Alexandros Mekras
Allgemein- und Viszeralchirurgie, SHG-Klinikum, Merzig , Deutschland

Dr. med. Ralf Uhlig
Klinik für Chirurgie, St.-Josef-Krankenhaus, Potsdam, Deutschland

Dr. med. Hagen Rudolph
Klinik für Allgemein- und Viszeralchirurgie, Klinikum Chemnitz gGmbH, Chemnitz, Deutschland

PD Dr. med. Burkhard Stoffels
Klinik für Allg.-, Viszeral- u. Unfallchirurgie, Heilig Geist-Krankenhaus GmbH, Köln, Deutschland
